# Supplementary material for: Reducibility of higher-order networks from dynamics
Source: Nat Commun. 2026 Jan 15;17:1551. doi: 10.1038/s41467-025-68273-4 (PMC12894711; doi:10.1038/s41467-025-68273-4)
Supplement: Supplementary file 1 — Supplementary Information [file 41467_2025_68273_MOESM1_ESM.pdf]

Supplementary Material: Reducibility of higher-order networks from dynamics

Maxime Lucas, Luca Gallo, Arsham Ghavasieh, Federico Battiston, and Manlio De Domenico

I. STRUCTURES WITH PROPORTIONAL LAPLACIANS

We expect structures where the Laplacians at each order are proportional, $\mathrm{L}^{(d)} \propto \mathrm{L}^{(d')}$, to be invariant under reduction, as detailed in Sections C and N. Here, we illustrate the corresponding flat cost function in Fig. S1, for the complete hypergraph and a triangular lattice. In those cases, the optimal order is lowest one, $d_{\mathrm{opt}} = 1$.

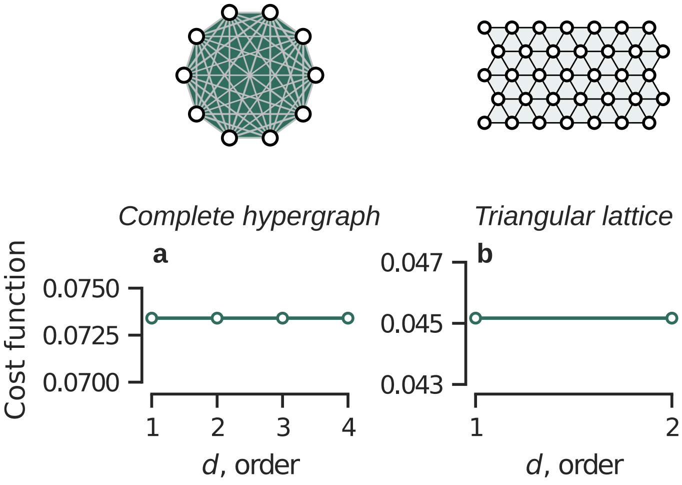

FIG. S1. **Hypergraphs with proportional Laplacians at each order have a flat cost function**. (a) Complete hypergraph, (b) triangular lattice flag complex. Parameters were set to 10 and 35 nodes, respectively with $d_{\max} = 4$ and 2, respectively.

II. ANALYTICAL DERIVATION OF THE HYPERRING CASE

Definition of the hyperring.—We consider a hyperring on N nodes, with periodic boundary conditions, including:

- Pairwise interactions of node i with first neighbor on each side: $(i, i+1) \mod N, \forall i = 1, \ldots, N$,
- Triplet interactions of node i with first neighbor on each side: $(i-1, i, i+1) \mod N, \forall i = 1, \ldots, N$,

so we have interactions of orders 1 and 2, with $d_{\max} = 2$. This structure is similar to the one considered in Ref. [1] and yields circulant matrices due to the rotational symmetry. This symmetry allows us to analytically derive the cost function in this case.

Circulant matrices.— A symmetric circulant matrix is a matrix of the form [2]:

$$\mathbf{A} = \begin{bmatrix} c_0 & c_1 & c_2 & \cdots & c_3 & c_2 & c_1 \\ c_1 & c_0 & c_1 & \cdots & c_4 & c_3 & c_2 \\ \vdots & \vdots & \vdots & \ddots & \vdots & \vdots & \vdots \\ c_2 & c_3 & c_4 & \cdots & c_1 & c_0 & c_1 \\ c_1 & c_2 & c_3 & \cdots & c_2 & c_1 & c_0 \end{bmatrix} \equiv \mathrm{circ}(c_0, c_1, c_2, \ldots, c_2, c_1), \tag{S1}$$

which can be defined by its first row. In general, circulant matrices are defined by N coefficients c_i, but for symmetric circulant matrices $c_j = c_{N-j}$ for $0 < j < \lfloor N - 1/2 \rfloor$. In addition, the sum of two circulant matrices is a circulant matrix. Because of this and of the rotational symmetry of the hyperring case, the adjacency, Laplacian, and density matrices are all (symmetric) circulant matrices. This makes them commute because they can be diagonalized simultaneously in the Fourier basis.

The eigenvalues of a symmetric circulant matrix are given by:

$$\lambda_k = \sum_{j=0}^{N-1} c_j e^{-2\pi i k j/N}, \tag{S2}$$

with $k = 0, \ldots, N-1$ and the symmetry $\lambda_k = \lambda_{N-k}$ for $0 < k < \lfloor N - 1/2 \rfloor$.

Multiorder Laplacian.— For convenience, we restate the following definitions from the main text. For a given hypergraph, the multiorder Laplacian is defined as:

$$\mathbf{L}^{[d]} = \sum_{\delta=1}^{d} \frac{1}{\langle K^{(\delta)} \rangle} \mathbf{L}^{(\delta)} \tag{S3}$$

where $\mathbf{L}^{(\delta)} = \mathbf{K}^{(\delta)} - \frac{1}{\delta}\mathbf{A}^{(d)}$is the Laplacian associated to hyperedges of order δ. For convenience, we also define the weights $w_\delta = 1/\langle K^{(\delta)} \rangle$.

Laplacian eigenvalues at order 1— We have $A_{ij}^{(1)} = 1$ if $|j - i| = 1$ but 0 otherwise, so the adjacency matrix is circulant and defined by $\mathbf{A}^{(1)} = \mathrm{circ}(0, 1, 0, \ldots, 0, 1)$. Each node is connected to its two nearest neighbors, so $K^{(1)} = 2$. The Laplacian is:

$$\mathbf{L}^{(1)} = \mathrm{circ}(2, -1, 0, \ldots, 0, -1), \tag{S4}$$

with eigenvalues

$$\lambda_k^{(1)} = 2 - 2\cos\left(\frac{2\pi k}{N}, \right), \tag{S5}$$

where we used the identity $\cos(x) = \frac{e^{ix} + e^{-ix}}{2}$. The associated multiorder Laplacian is simply rescaled by $K^{(1)}$, $\mathbf{L}^{[1]} = \frac{1}{2}\mathbf{L}^{(1)}$, and its eigenvalues are

$$\lambda_k^{[1]} = 1 - \cos\left(\frac{2\pi k}{N}\right). \tag{S6}$$

Laplacian eigenvalues at order 2— Each node i participates in 3 hyperedges:

$$(i-2, i-1, i), \quad (i-1, i, i+1), \quad (i, i+1, i+2), \tag{S7}$$

so $K^{(2)} = 3$. Additionally, $A_{ij}^{(2)} = 2$ if $|j - i| = 1$ but 1 if $|j - i| = 2$ and 0 otherwise, which means that it is circulant and defined by $\mathbf{A}^{(2)} = \mathrm{circ}(0, 2, 1, 0, \ldots, 0, 1, 2)$. The Laplacian is:

$$\mathbf{L}^{(2)} = \mathrm{circ}(3, -1, -\frac{1}{2}, 0, \ldots, 0, -\frac{1}{2}, -1), \tag{S8}$$

with eigenvalues:

$$\lambda_k^{(2)} = 3 - 2\cos\left(\frac{2\pi k}{N}\right) - \cos\left(\frac{4\pi k}{N}\right). \tag{S9}$$

The associated multiorder Laplacian is defined as $\mathbf{L}^{[2]} = \frac{1}{2}\mathbf{L}^{(1)} + \frac{1}{3}\mathbf{L}^{(2)}$. Because $\mathbf{L}^{(1)}$ and $\mathbf{L}^{(2)}$ are circulant, they commute, and the eigenvalues of their sum are the sum of their eigenvalues—this is an important simplification that does not hold for generic hypergraphs. Thus, the eigenvalues are given by

$$\lambda_k^{[2]} = w_1 \lambda_k^{(1)} + w_2 \lambda_k^{(2)}, \tag{S10}$$

$$= \left[1 - \cos\left(\frac{2\pi k}{N}\right)\right] + \left[1 - \frac{2}{3}\cos\left(\frac{2\pi k}{N}\right) - \frac{1}{3}\cos\left(\frac{4\pi k}{N}\right)\right], \tag{S11}$$

$$= 2 - \frac{5}{3}\cos\left(\frac{2\pi k}{N}\right) - \frac{1}{3}\cos\left(\frac{4\pi k}{N}\right). \tag{S12}$$

Note that for all the above spectra, the mode $k = 0$ always corresponds to the 0 eigenvalue. This corresponds to the conservation of mass by the diffusion. We now have explicit expressions for all Laplacian eigenvalues, which we plot in Fig. 3a.

Density matrices and their spectra.— The multiorder density matrix up to order d is defined in the main text as

$$\boldsymbol{\rho}_{\tau'}^{[d]} = \frac{e^{-\tau' \mathbf{L}^{[d]}}}{Z^{[d]}}, \quad Z^{[d]} = \mathrm{Tr}(e^{-\tau' \mathbf{L}^{[d]}}), \tag{S13}$$

where we denote $\tau' = \tau'(d) = \frac{d_{\max}}{d}\tau$ (Eq. (7)) to avoid cluttering the notation, which makes the dependence in d become implicit in the notation. Because the Laplacians are circulant, so are the density matrices. The eigenvalues $\mu_k^{[d]}$ of the density matrix can be expressed in terms of those of the corresponding Laplacian matrix for $k = 0, \ldots, N$ [3]

$$\mu_k^{[d]} = \frac{e^{-\tau' \lambda_k^{[d]}}}{\sum_j e^{-\tau' \lambda_j^{[d]}}}, \tag{S14}$$

which depend on the diffusion τ, contrary to the Laplacian eigenvalues. The following symmetry follows from the same symmetry in the Laplacian eigenvalues: $\mu_k = \mu_{N-k}$ for $0 < k < \lfloor (N-1)/2 \rfloor$. Additionally, they sum to 1, $\sum_k \mu_k^{[d]} = 1$. Since we derived explicit expressions for the Laplacian eigenvalues above, we have explicit expressions for the density matrices, which we do not write down because they are not informative, but we plot the spectra in Fig. 3b.

Physically, this density matrix is a normalized propagator of the diffusion at time τ', its eigenvectors are diffusion modes, and its eigenvalues are the weights (probabilities) associated with these modes. The Laplacian eigenvalues determine the time scale of these modes: fast modes (large $\lambda_k^{[d]}$) decay faster whereas slow modes (low $\lambda_k^{[d]}$) decay slower. Consequently, at short diffusion times, their corresponding weight $\mu_k^{[d]}$ is similar regardless of the magnitude of the eigenvalue, but at longer diffusion times, weight concentrates on slow modes and fast modes have little weight because they have decayed. In limiting cases:

- At very short times, $\tau \to 0$: $\mu_k^{[d]} \to 1/N$, which means that all modes contribute equally because no diffusion has occurred yet. Formally, this is because we have $Z^{[d]} \to N$, $\boldsymbol{\rho}_{\tau'}^{[d]} \to \mathbf{Id}/N$.
- At very long times, $\tau \to +\infty$: $\mu_0^{[d]} \to 1$ whereas $\mu_{>0}^{[d]} \to 0$ because all eigenmodes have decayed except for constant mode $k = 0$ with Laplacian eigenvalue 0. Formally, $Z^{[d]} \to 1$.

This behavior and physical intuition of the effect of τ described above are valid for any hypergraph, because Eq. (S14) holds in general, even in cases where the Laplacian eigenvalues are different and cannot be derived explicitly. In fact, it has already been discussed for pairwise graphs, for example in Ref. [3]. The effect of d will instead depend on how the truncated Laplacian spectra change as more orders are truncated.

Kullback-Leibler Divergence.— The KL divergence can be written as

$$D_{\mathrm{KL}}\left(\boldsymbol{\rho}_{\tau}^{[d_{\max}]} | \boldsymbol{\rho}_{\tau'}^{[d]}\right) = \mathrm{Tr}\left[\boldsymbol{\rho}_{\tau}^{[d_{\max}]}\left(\log \boldsymbol{\rho}_{\tau}^{[d_{\max}]} - \log \boldsymbol{\rho}_{\tau'}^{[d]}\right)\right] \tag{S15}$$

$$= \sum_k \mu_k^{[d_{\max}]} \log\left(\frac{\mu_k^{[d_{\max}]}}{\mu_k^{[d]}}\right) \tag{S16}$$

where the first line is the definition from the main text, and the second line uses the fact that the two density matrices commute because they are circulant. Because of this, the KL divergence reduces to one of distributions where the distributions are those of the eigenvalues, $p_k = \mu_k^{[d_{\max}]}$ and $q_k = \mu_k^{[d]}$. For general hypergraphs, the cross-entropy term $\mathrm{Tr}\left[\boldsymbol{\rho}_{\tau}^{[d_{\max}]} \log \boldsymbol{\rho}_{\tau'}^{[d]}\right]$ cannot be simplified this way because the two matrices do not commute.

In the present case, the logarithm can be rewritten

$$\log\left(\frac{\mu_k^{[d_{\max}]}}{\mu_k^{[d]}}\right) = \log\left(\frac{e^{-\tau\lambda_k^{[d_{\max}]}}}{e^{-\tau'\lambda_k^{[d]}}} \frac{Z^{[d]}}{Z^{[d_{\max}]}}\right), \tag{S17}$$

$$= \left(\tau'\lambda_k^{[d]} - \tau\lambda_k^{[d_{\max}]}\right) + \log\left(\frac{Z^{[d]}}{Z^{[d_{\max}]}}\right), \tag{S18}$$

$$= \tau\left(\frac{d_{\max}}{d}\lambda_k^{[d]} - \lambda_k^{[d_{\max}]}\right) + \log\left(\frac{Z^{[d]}}{Z^{[d_{\max}]}}\right), \tag{S19}$$

where we used the definition of τ' in the last line. We can then rewrite the divergence as

$$D_{\mathrm{KL}}\left(\boldsymbol{\rho}_\tau^{[d_{\max}]}|\boldsymbol{\rho}_{\tau'}^{[d]}\right) = \sum_k \frac{e^{-\tau\lambda_k^{[d_{\max}]}}}{Z^{[d_{\max}]}}\left[\tau\left(\frac{d_{\max}}{d}\lambda_k^{[d]} - \lambda_k^{[d_{\max}]}\right) + \log\left(\frac{Z^{[d]}}{Z^{[d_{\max}]}}\right)\right], \tag{S20}$$

$$= \tau\sum_k \frac{e^{-\tau\lambda_k^{[d_{\max}]}}}{Z^{[d_{\max}]}}\left(\frac{d_{\max}}{d}\lambda_k^{[d]} - \lambda_k^{[d_{\max}]}\right) + \log\left(\frac{Z^{[d]}}{Z^{[d_{\max}]}}\right), \tag{S21}$$

$$= \tau\,\mathbb{E}_{\rho^{[d_{\max}]}}\left[\frac{d_{\max}}{d}\lambda_k^{[d]} - \lambda_k^{[d_{\max}]}\right] + \log\left(\frac{Z^{[d]}}{Z^{[d_{\max}]}}\right), \tag{S22}$$

where the second line uses the fact that the $\mu_k^{[d_{\max}]}$ sum to 1, and where $\mathbb{E}_{\rho^{[d_{\max}]}}[\cdot]$ denotes the expectation over the spectrum of $\boldsymbol{\rho}_\tau^{[d_{\max}]}$, using its eigenvalues $\mu_k^{[d_{\max}]}$ as weights:

$$\mathbb{E}_{\rho^{[d_{\max}]}}[\lambda_k] = \sum_{k=0}^{N-1}\frac{e^{-\tau\lambda_k^{[d_{\max}]}}}{Z^{[d_{\max}]}}\cdot\lambda_k = \sum_{k=0}^{N-1}\mu_k^{[d_{\max}]}\cdot\lambda_k. \tag{S23}$$

Since the Laplacians commute, the eigenvalues $\lambda_k^{[d]}$ of the truncated system can be expressed in terms of the eigenvalues of each order $\lambda_k^{(d)}$ as $\lambda_k^{[d]} = \sum_{\delta=1}^{d} w_\delta\lambda_k^{(\delta)}$. We can then further develop the first term of the KL divergence:

$$\frac{d_{\max}}{d}\lambda_k^{[d]} - \lambda_k^{[d_{\max}]} = (\frac{d_{\max}}{d} - 1)\sum_{\delta=1}^{d} w_\delta\lambda_k^{(\delta)} - \sum_{\delta=d+1}^{d_{\max}} w_\delta\lambda_k^{(\delta)}, \tag{S24}$$

so that expectation can then be rewritten as

$$\mathbb{E}_{\rho^{[d_{\max}]}}\left[\frac{d_{\max}}{d}\lambda_k^{[d]} - \lambda_k^{[d_{\max}]}\right] = (\frac{d_{\max}}{d} - 1)\sum_{\delta=1}^{d} w_\delta\mathbb{E}_{\rho^{[d_{\max}]}}\left[\lambda_k^{(\delta)}\right] - \sum_{\delta=d+1}^{d_{\max}} w_\delta\mathbb{E}_{\rho^{[d_{\max}]}}\left[\lambda_k^{(\delta)}\right], \tag{S25}$$

or equivalently,

$$\mathbb{E}_{\rho^{[d_{\max}]}}\left[\frac{d_{\max}}{d}\lambda_k^{[d]} - \lambda_k^{[d_{\max}]}\right] = (\frac{d_{\max}}{d} - 1)\mathbb{E}_{\rho^{[d_{\max}]}}\left[\lambda_k^{[d]}\right] - \mathbb{E}_{\rho^{[d_{\max}]}}\left[\lambda_k^{\lfloor\delta\rfloor}\right], \tag{S26}$$

where we denote $\lambda_k^{\lfloor d\rfloor} = \sum_{\delta=d+1}^{d_{\max}} w_\delta\lambda_k^{(\delta)}$ the eigenvalues of the hypergraph composed of the truncated orders of the original hypergraph (the "dual" of the truncated hypergraph), using the symbol $\lfloor\cdot\rfloor$. The final expression for the KL divergence is thus

$$D_{\mathrm{KL}}\left(\boldsymbol{\rho}_\tau^{[d_{\max}]}|\boldsymbol{\rho}_{\tau'}^{[d]}\right) = \tau\left[(\frac{d_{\max}}{d} - 1)\mathbb{E}_{\rho^{[d_{\max}]}}\left[\lambda_k^{[d]}\right] - \mathbb{E}_{\rho^{[d_{\max}]}}\left[\lambda_k^{\lfloor\delta\rfloor}\right]\right] + \log\left(\frac{Z^{[d]}}{Z^{[d_{\max}]}}\right). \tag{S27}$$

This expression of the information loss has a structure similar to others derived for pairwise networks [3] and we can interpret it. The *first term* (1) is proportional to τ and accounts for contributions due to *spectral differences* between the full hypergraph and its reduced form. It is composed of (1a) a term quantifying the average Laplacian eigenvalue of the truncated system sampled from the associated modes under the full system diffusion. The $(\frac{d_{\max}}{d} - 1)$ pre-factor accounts for the rescaling of τ and vanishes when $d = d_{\max}$. Then (1b) accounts for the contributions of the orders discarded in the truncated system ($d < \delta \leq d_{\max}$). Finally, the *second term* (2) is a log-correction of the spread of the two density matrix spectra.

Effect of order d.— Let us fix the diffusion time. When the truncated system is equal to the full system, $d = d_{\max}$, the information loss vanishes because both terms (1) and (2) vanish, as expected.

Effect of diffusion time τ.— Let us fix the order d.

- At very short times $\tau \to 0$: $D_{\mathrm{KL}}\left(\boldsymbol{\rho}_\tau^{[d_{\max}]}|\boldsymbol{\rho}_{\tau'}^{[d]}\right) \to 0$. Both models are equivalent because no diffusion has occurred yet. Formally, this is because we know that $\mu_k^{[d]} \to 1/N$ so that $\mathbb{E}_{\rho^{[d_{\max}]}}[\lambda_k] \to \frac{1}{N}\sum_k \lambda_k$ is simply the average eigenvalue of the spectrum, and terms (1) and (2) vanish.

- At very long times, $\tau \to \infty$: $D_{\mathrm{KL}}\left(\boldsymbol{\rho}_{\tau}^{[d_{\max}]} | \boldsymbol{\rho}_{\tau'}^{[d]}\right) \to 0$. Both models are equivalent because all modes have had time to decay, except for the common uniform mode. This is because all modes decay except for $k = 0$ which has $\lambda_0^{[d_{\max}]} = 0$ so that its exponential is constantly 1. So, $\mathbb{E}_{\rho^{[d_{\max}]}}[\lambda_k] \to \lambda_0 = 0$. Because of this, term (1) vanishes, and term (2) does too because the partition functions $\to 1$.

The fact that the information loss vanishes at these two extreme diffusion times indicates that they are not informative scales at which to probe the system. Instead, diffusion times based on the times scales of the system (i.e. the Laplacian eigenvalues) are more meaningful (see main text). At those informative time scales:

- At short times $\tau \sim \tau_{\mathrm{short}}$: the expectations $\mathbb{E}_{\rho^{[d_{\max}]}}[\lambda_k] = \sum_{k=0}^{N-1} \mu_k^{[d_{\max}]} \cdot \lambda_k$ weigh all Laplacian eigenvalues similarly, so that the fast modes (high eigenvalues) dominate, which correspond to local patterns (localized eigenvectors).

- At long times $\tau \sim \tau_{\mathrm{long}}$: here, the expectations are dominated by the slow modes (low eigenvalues) which correspond to global patterns (uniform eigenvectors)

Complexity term.— In the main text, we define the complexity of the model up to order d as the Kullback-Leibler divergence compared to the isolated hypergraph case (no hyperedges):

$$C(\boldsymbol{\rho}_{\tau'}^{[d]}) = D_{\mathrm{KL}}\left(\boldsymbol{\rho}_{\tau'}^{[d]} \,|\, \boldsymbol{\rho}_{\mathrm{iso}}\right) = \log N - S_{\tau'}^{[d]}, \tag{S28}$$

where $S_{\tau'}^{[d]}$ is the von Neumann entropy is defined as:

$$S_{\tau'}^{[d]} = -\operatorname{Tr}\left(\boldsymbol{\rho}_{\tau'}^{[d]} \log \boldsymbol{\rho}_{\tau'}^{[d]}\right) = \tau' \, \mathbb{E}_{\rho^{[d]}}\left[\lambda_k^{[d]}\right] + \log Z^{[d]}, \tag{S29}$$

where $\mathbb{E}_{\rho^{[d]}}\left[\lambda_k^{[d]}\right]$ is the expectation of the eigenvalues order d, weighted by the eigenvalues of the diffusion in the truncated hypergraph. Hence, the complexity becomes:

$$C(\boldsymbol{\rho}_{\tau'}^{[d]}) = \log\left(\frac{N}{Z^{[d]}}\right) - \tau \frac{d_{\max}}{d} \, \mathbb{E}_{\rho^{[d]}}\left[\lambda_k^{[d]}\right], \tag{S30}$$

which is valid for any hypergraph.

- At very short times $\tau \to 0$, we have $C(\boldsymbol{\rho}_{\tau'}^{[d]}) \to 0$. The complexity is null because no diffusion occurs.

- At very long times, $\tau \to \infty$, $C(\boldsymbol{\rho}_{\tau'}^{[d]}) \to \log N$.

Cost function.— The total cost function of the truncated hypergraph at order d is given by the sum of the two previously derived terms:

$$\mathcal{L}\left(\boldsymbol{\rho}_{\tau}^{[d_{\max}]} | \boldsymbol{\rho}_{\tau'}^{[d]}\right) = D_{\mathrm{KL}}\left(\boldsymbol{\rho}_{\tau}^{[d_{\max}]} \,|\, \boldsymbol{\rho}_{\tau'}^{[d]}\right) + C(\boldsymbol{\rho}_{\tau'}^{[d]}). \tag{S31}$$

$$= \tau\left[\left(\frac{d_{\max}}{d} - 1\right) \mathbb{E}_{\rho^{[d_{\max}]}}\left[\lambda_k^{[d]}\right] - \mathbb{E}_{\rho^{[d_{\max}]}}\left[\lambda_k^{\lfloor\delta\rfloor}\right] + \log\left(\frac{Z^{[d]}}{Z^{[d_{\max}]}}\right)\right] - \tau \frac{d_{\max}}{d} \mathbb{E}_{\rho^{[d]}}\left[\lambda_k^{[d]}\right] + \log\left(\frac{N}{Z^{[d]}}\right). \tag{S32}$$

We can reorder the terms as follows:

$$\mathcal{L}\left(\boldsymbol{\rho}_{\tau}^{[d_{\max}]} | \boldsymbol{\rho}_{\tau'}^{[d]}\right) = \tau\left[\left(\frac{d_{\max}}{d} - 1\right) \mathbb{E}_{\rho^{[d_{\max}]}}\left[\lambda_k^{[d]}\right] - \mathbb{E}_{\rho^{[d_{\max}]}}\left[\lambda_k^{\lfloor\delta\rfloor}\right] - \frac{d_{\max}}{d} \mathbb{E}_{\rho^{[d]}}\left[\lambda_k^{[d]}\right]\right] + \log\left(\frac{Z^{[d]}}{Z^{[d_{\max}]}}\right) + \log\left(\frac{N}{Z^{[d]}}\right). \tag{S33}$$

Interpretation and limit cases.—
Effect of diffusion time τ at fixed order d.—

- $\tau \to 0$: As describe above, both the information loss and the complexity are null. Hence,

$$\mathcal{L}\left(\boldsymbol{\rho}_{\tau}^{[d_{\max}]}|\boldsymbol{\rho}_{\tau'}^{[d]}\right) \to 0 \tag{S34}$$

In this case, all quantities vanish and do not depend on d. The cost function is flat and one should select the smallest order. This is because at $\tau = 0$, no diffusion has occurred, so that all hypergraphs are equivalent from the point of view of diffusion. There is no information loss and no complexity. In a sense, this case is trivial and does not give us any information because the diffusion time is too small.

- $\tau \to \infty$: As describe above, the information loss is null and the complexity tends to $\log N$. Hence,

$$\mathcal{L}\left(\boldsymbol{\rho}_{\tau}^{[d_{\max}]}|\boldsymbol{\rho}_{\tau'}^{[d]}\right) \to \log N \tag{S35}$$

In this case, we let diffusion to go infinitely long and all eigenmodes have time to vanish except for the constant mode $k = 0$ which corresponds to the 0 eigenvalue and the $(1, ..., 1)$ eigenmode, This corresponds to when diffusion has reached equilibrium over the whole hypergraph and is not informative either.

III. RANDOM SYNTHETIC STRUCTURES: EFFECT OF DIFFUSION TIME AND DENSITY

In this section, we illustrate the effect of diffusion time τ and density on the cost function and reducibility of random synthetic structures.

Figure S3 shows the cost function of a random simplicial complex for a wide range of diffusion times. We see that the structure is slightly reducible at all diffusion times, except at very large diffusion times $\tau > \tau_{\text{long}}$ where it becomes fully irreducible. Figure S3 shows the cost function of a random simplicial complex for three increasing values of density, at a fixed short diffusion time τ_{short}. Results indicate that the random simplicial complex remains slightly reducible across densities at that time scale. Exploring a grid of both parameters (τ, density), Fig. S4 shows results consistent with the above observations.

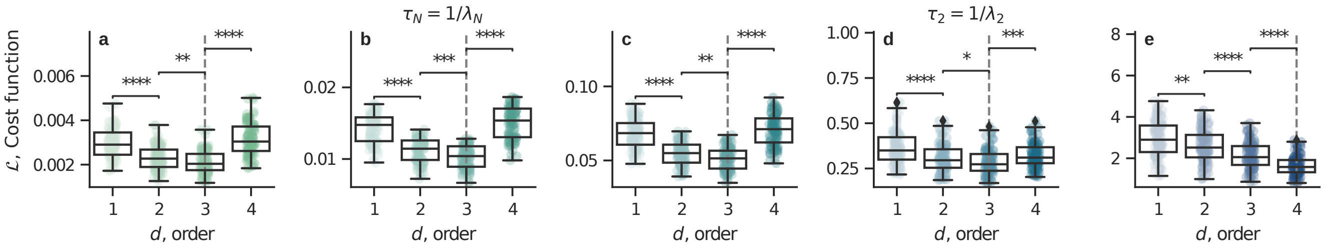

FIG. S2. **Effect of** τ. We show the cost function for a random simplicial complex, as a function of order d, for increasing values of the base diffusion time τ (from left to right). The second and fourth values are $\tau_{\text{short}} = 1/\lambda_{\max}$ and $\tau_{\text{long}} = 1/\lambda_{\min}$ based on eigenvalues of the multiorder Laplacian matrix. Other values are chosen to be evenly spaced in logarithmic scale. The minimum of the cost function is indicated by the vertical line. Parameters were set to $N = 100$ nodes and wiring probabilities $p_d = 50/N^d$ at order d with $d_{\max} = 4$. Stars indicate a statistically significant difference between two distributions (t-test, s stars indicate $p < 10^{-s}$).

IV. EMPIRICAL DATASETS

A. Cost functions

In Figs. S5 and S6, we show the cost functions across orders for each of the 60 empirical datasets, and mark the corresponding optimal order (vertical dashed line).

B. Reducibility at short and long diffusion times

In this section, we illustrate the effect of diffusion time τ and density on the cost function and reducibility of random synthetic structures.

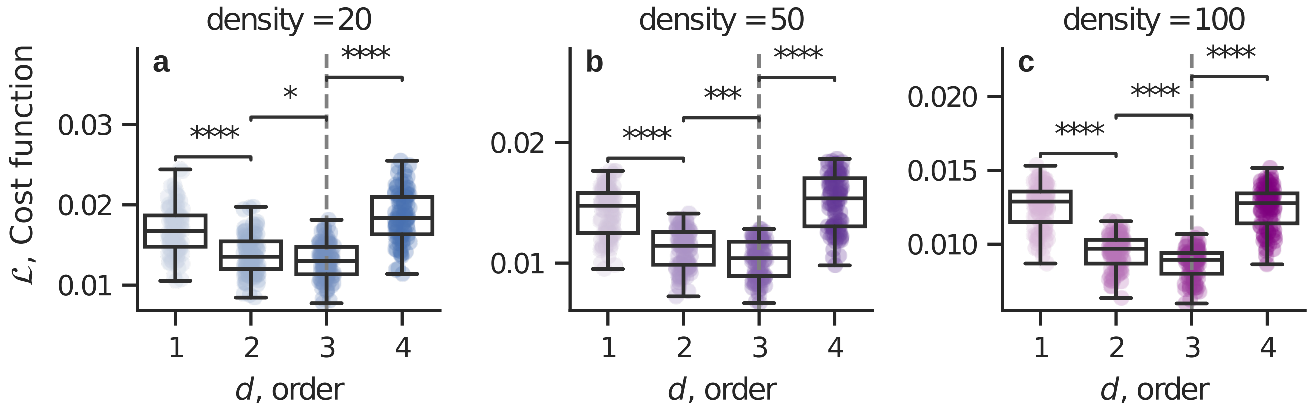

FIG. S3. **Effect of density**. We show the cost function for a random simplicial complex, as a function of order d, for increasing values of the density coefficient (from left to right), i.e., wiring probabilities are $p_d = 20/N^d$, $p_d = 50/N^d$, and $p_d = 100/N^d$ at order d. The minimum of the cost function is indicated by the vertical line. Parameters were set to $N = 100$ nodes and $d_{\max} = 4$, with $\tau = 1/\lambda_{\max}$. Stars indicate a statistically significant difference between two distributions (t-test, s stars indicate $p < 10^{-s}$).

C. Impact of structural metrics on reducibility

To add to the results shown in Fig. 6, we show how additional metrics correlate with reducibility (Fig. S8a-f), and how all metrics correlate between themselves (Fig. S8g).

To add to the results shown in Fig. 7, we show how additional metrics are impacted by the three randomizing strategies (Fig. S9).

V. ALTERNATIVE DEFINITIONS

In the main text, we have argued that the KL divergence Eq. (21) and the entropy Eq. (23) are meaningful and natural choices to define the information loss and the model complexity. Nonetheless, unlike in the original minimal message length formalism [4], there is no unique self-consistent way to define these two terms (as we have discussed) and they can be defined independently. We now give alternative definitions for these terms using a symmetric and bounded version of the KL divergence: the Jensen-Shannon (JS) divergence. The information loss can thus be defined by

$$D_{\mathrm{JS}}\left(\boldsymbol{\rho}_\tau^{[d_{\max}]}|\boldsymbol{\rho}_{\tau'}^{[d]}\right) = D_{\mathrm{KL}}\left(\boldsymbol{\rho}_\tau^{[d_{\max}]}|\boldsymbol{\rho}_\tau^{M}\right) + D_{\mathrm{KL}}\left(\boldsymbol{\rho}_{\tau'}^{[d]}|\boldsymbol{\rho}_\tau^{M}\right), \tag{S36}$$

which is bounded in $[0, 1]$, and where we denote the mixture matrix $\boldsymbol{\rho}_\tau^M = \left(\boldsymbol{\rho}_\tau^{[d_{\max}]} + \boldsymbol{\rho}_{\tau'}^{[d]}\right)/2$. We can then also rescale the original model complexity C to be bounded in the same interval:

$$\tilde{C}(\boldsymbol{\rho}_{\tau'}^{[d]}) = C(\boldsymbol{\rho}_{\tau'}^{[d]})/\log N = 1 - S_{\tau'}^{[d]}/\log N. \tag{S37}$$

Other definitions could also be possible, and dynamical processes other than diffusion too. As long as the quantities are well defined, the choice will depend both on the context, specific applications, and on how informative the results are in practice. For example, the alternative definitions Eqs. (S36) and (S37) result similar reducibility for the synthetic cases (Fig. S10) but in irreducibility for almost all empirical datasets, and all diffusion times and hence is not very informative (Fig. S11).

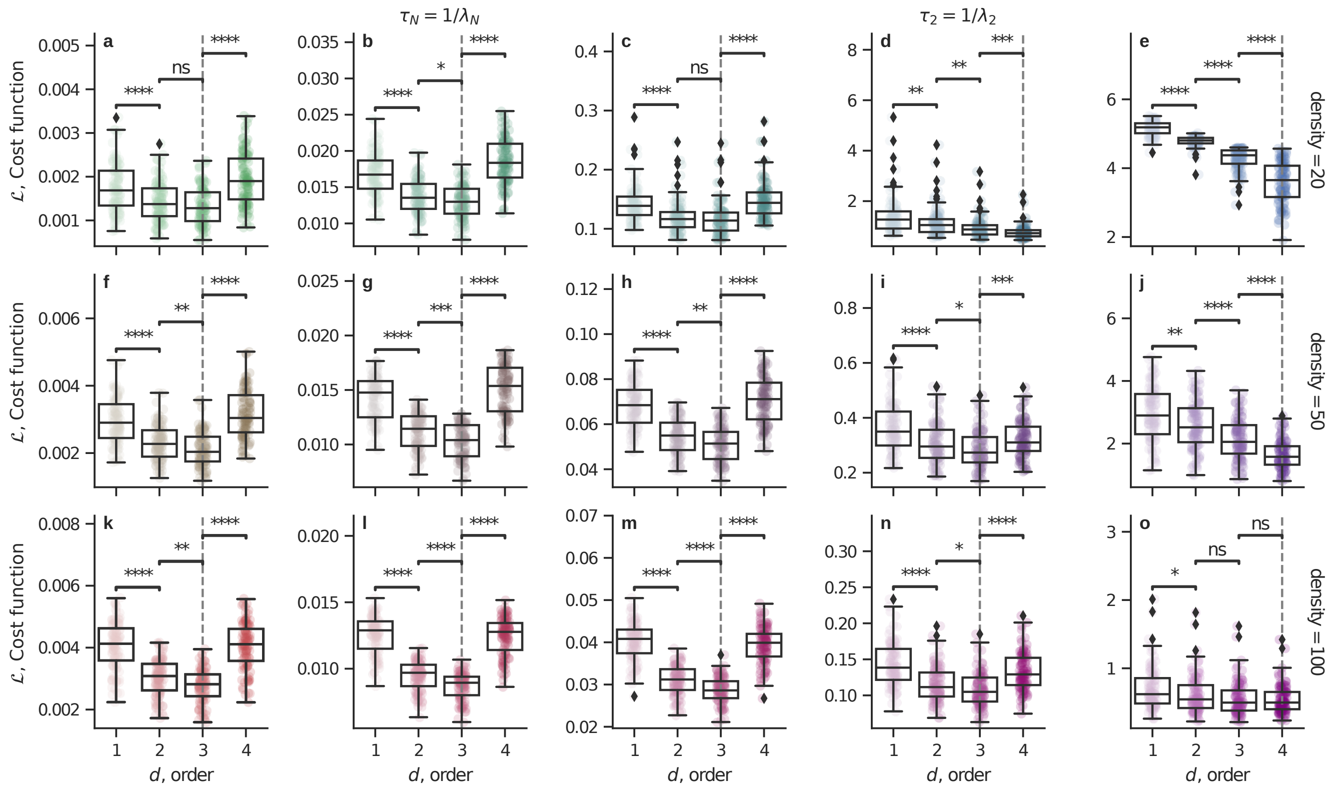

FIG. S4. **Effect of density and diffusion time.** We show the cost function for a random simplicial complex, as a function of order d, for increasing values of the base diffusion time τ (from left to right) and density (from top to bottom). The second and fourth values of diffusion time are $\tau_{\text{short}} = 1/\lambda_{\max}$ and $\tau_{\text{long}} = 1/\lambda_{\min}$ based on eigenvalues of the multiorder density matrix. Other values are chosen to be evenly spaced in logarithmic scale. The minimum of the cost function is indicated by the vertical line. Parameters were set to $N = 100$ nodes and wiring probabilities are $p_d = 20/N^d$, $p_d = 50/N^d$, and $p_d = 100/N^d$ at order d. with $d_{\max} = 4$. Stars indicate a statistically significant difference between two distributions (t-test, s stars indicate $p < 10^{-s}$, "ns" is non-significant).

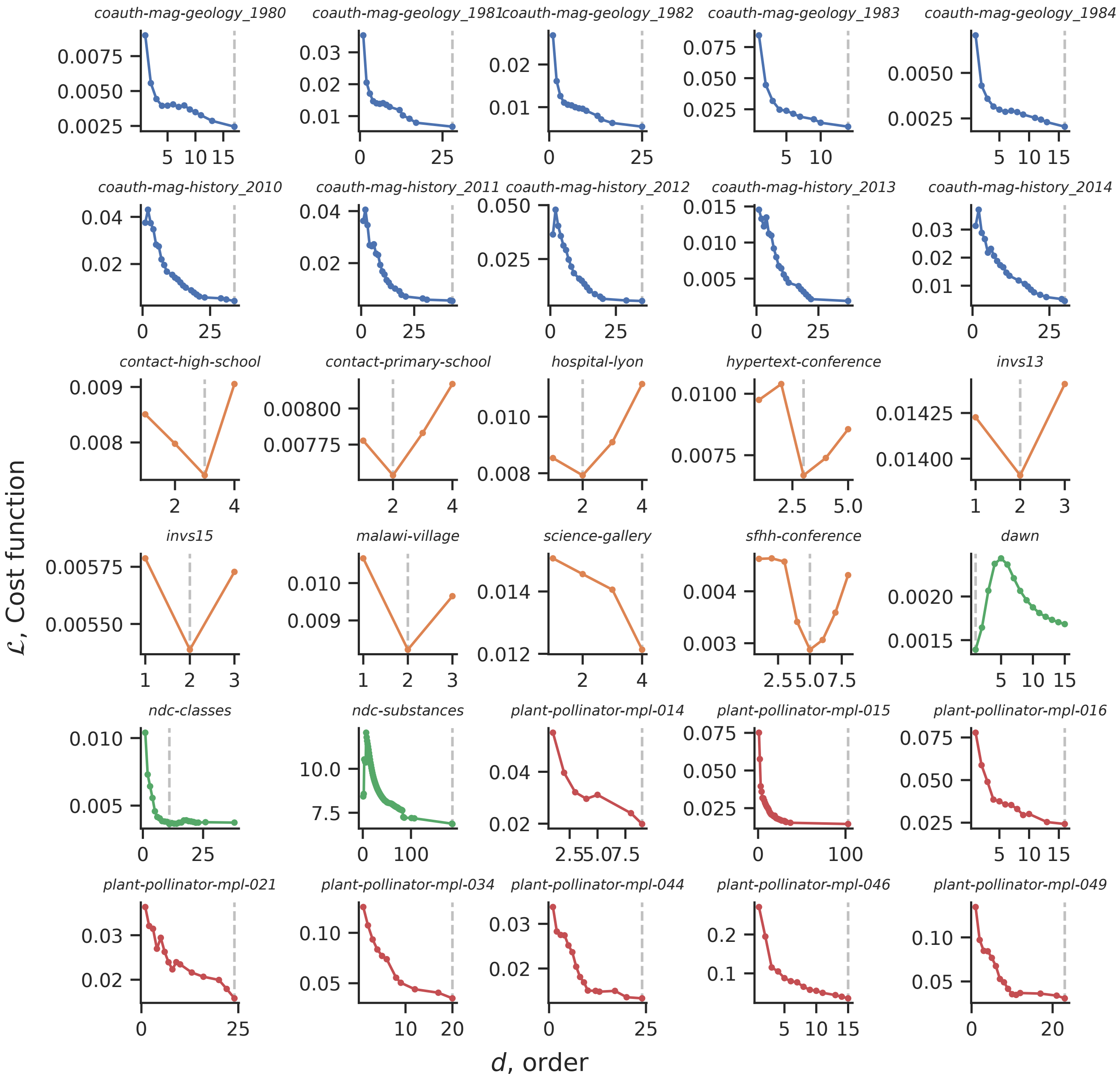

FIG. S5. **Cost function as a function of the order for all 60 empirical datasets (1-30)**. Vertical lines indicate the optimal order in each case. Note the variety of shapes of those cost function curves.

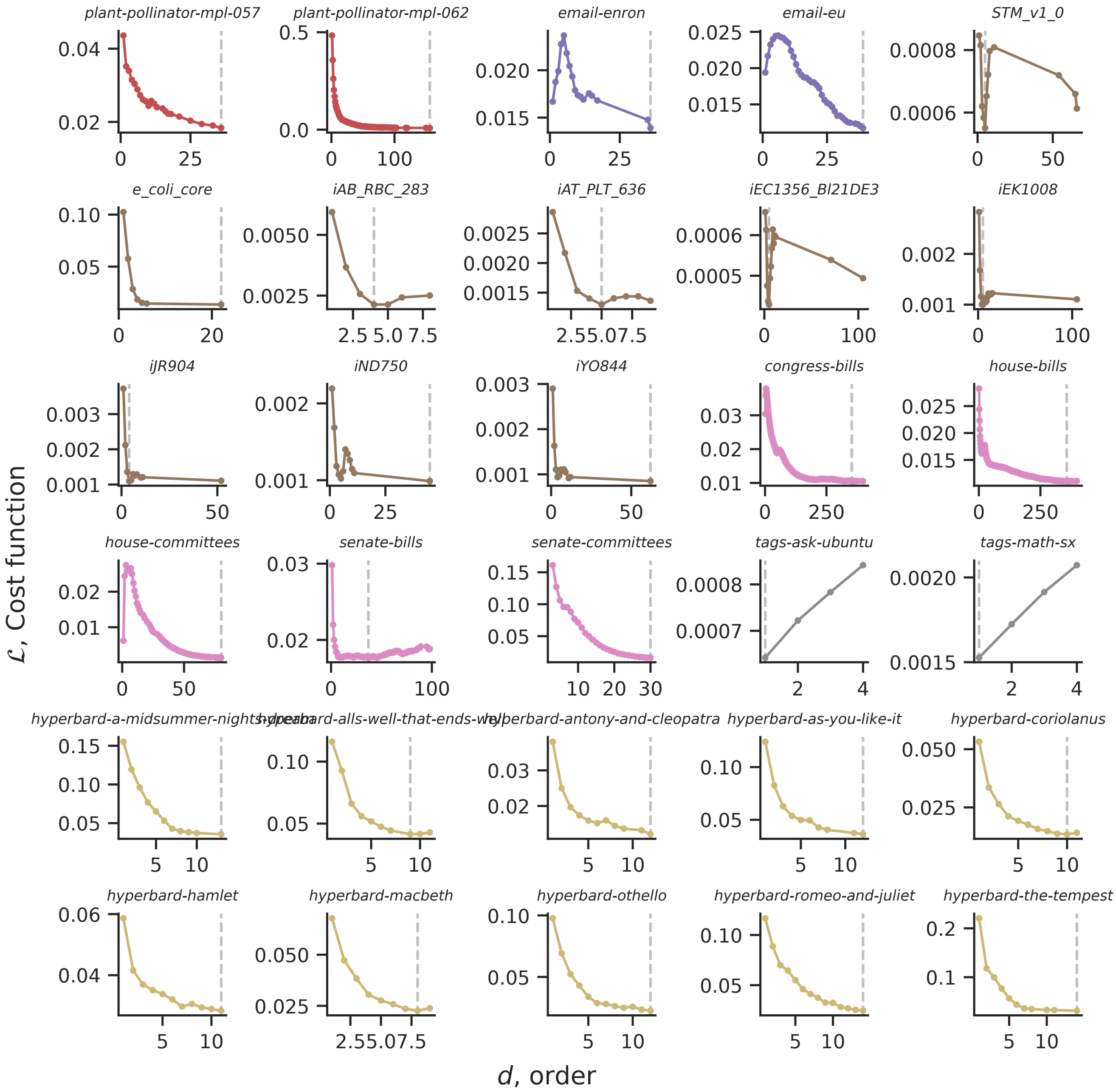

FIG. S6. **Cost function as a function of the order for all 60 empirical datasets (30-60)**. Vertical lines indicate the optimal order in each case. Note the variety of shapes of those cost function curves.

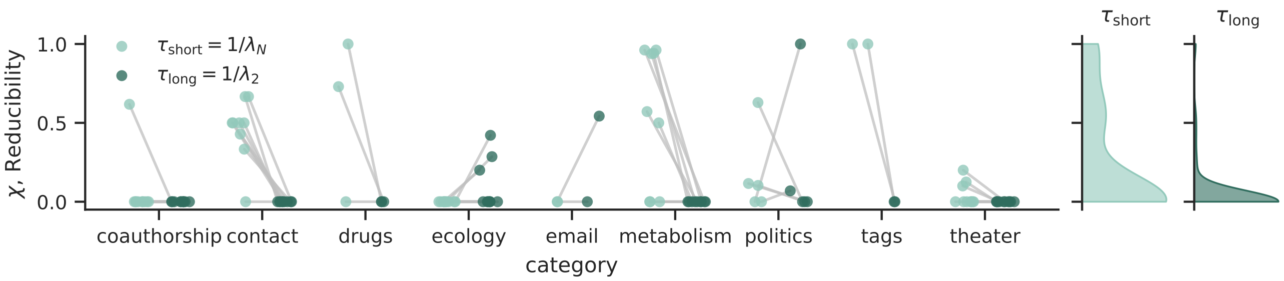

FIG. S7. **Sixty empirical datasets show different levels of reducibility**. We show the reducibility χ for all 60 datasets for the short diffusion time $\tau_{\rm short} = 1/\lambda_{\rm max}$ (light green, same data as in Fig. 5d), and a long $\tau_{\rm long} = 1/\lambda_{\rm min}$ (dark green). The overall distributions associated with each τ are shown at the far right.

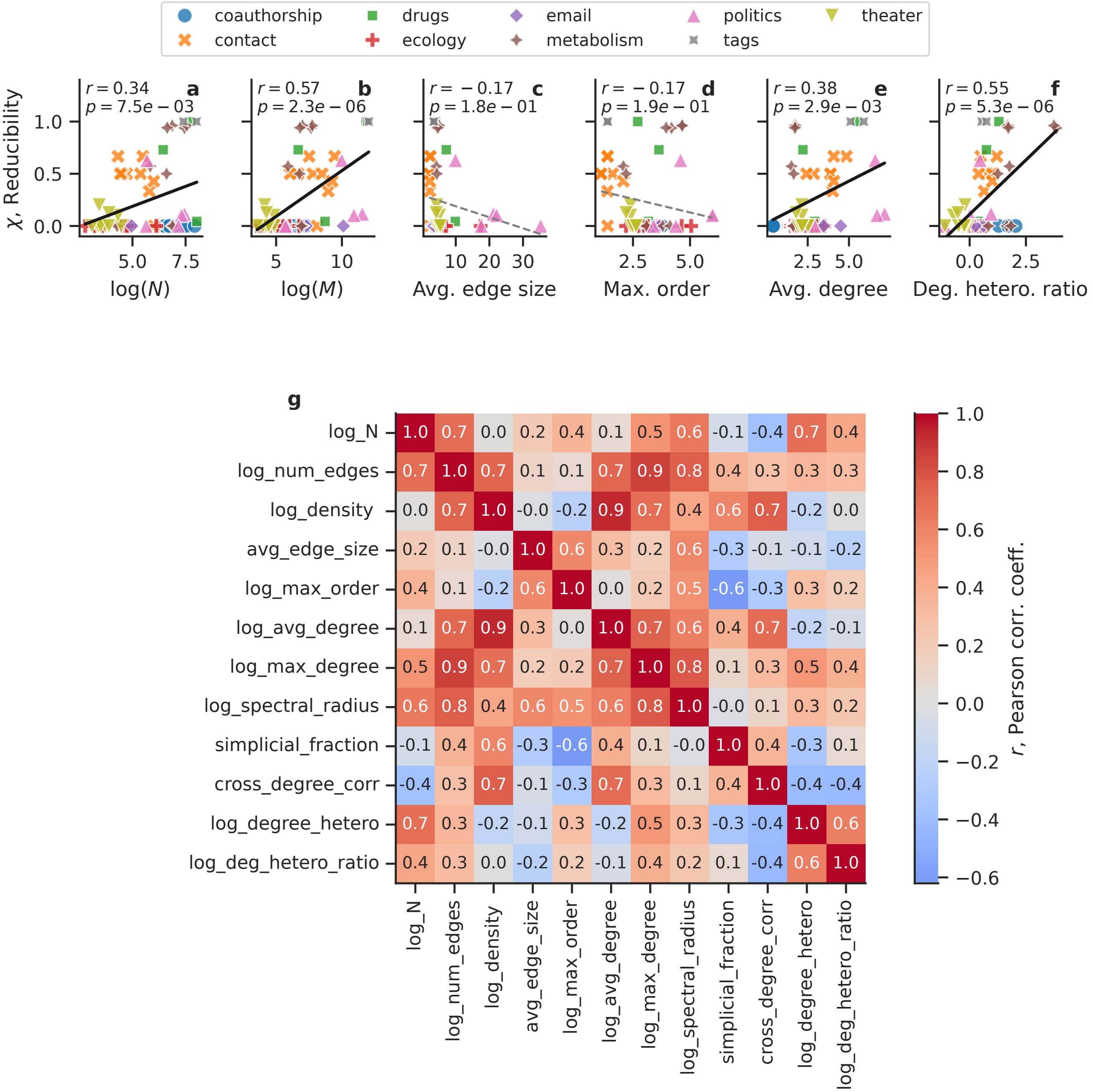

	log_N	log_num_edges	log_density	avg_edge_size	log_max_order	log_avg_degree	log_max_degree	log_spectral_radius	simplicial_fraction	cross_degree_corr	log_degree_hetero	log_deg_hetero_ratio
log_N	1.0	0.7	0.0	0.2	0.4	0.1	0.5	0.6	-0.1	-0.4	0.7	0.4
log_num_edges	0.7	1.0	0.7	0.1	0.1	0.7	0.9	0.8	0.4	0.3	0.3	0.3
log_density	0.0	0.7	1.0	-0.0	-0.2	0.9	0.7	0.4	0.6	0.7	-0.2	0.0
avg_edge_size	0.2	0.1	-0.0	1.0	0.6	0.3	0.2	0.6	-0.3	-0.1	-0.1	-0.2
log_max_order	0.4	0.1	-0.2	0.6	1.0	0.0	0.2	0.5	-0.6	-0.3	0.3	0.2
log_avg_degree	0.1	0.7	0.9	0.3	0.0	1.0	0.7	0.6	0.4	0.7	-0.2	-0.1
log_max_degree	0.5	0.9	0.7	0.2	0.2	0.7	1.0	0.8	0.1	0.3	0.5	0.4
log_spectral_radius	0.6	0.8	0.4	0.6	0.5	0.6	0.8	1.0	-0.0	0.1	0.3	0.2
simplicial_fraction	-0.1	0.4	0.6	-0.3	-0.6	0.4	0.1	-0.0	1.0	0.4	-0.3	0.1
cross_degree_corr	-0.4	0.3	0.7	-0.1	-0.3	0.7	0.3	0.1	0.4	1.0	-0.4	-0.4
log_degree_hetero	0.7	0.3	-0.2	-0.1	0.3	-0.2	0.5	0.3	-0.3	-0.4	1.0	0.6
log_deg_hetero_ratio	0.4	0.3	0.0	-0.2	0.2	-0.1	0.4	0.2	0.1	-0.4	0.6	1.0

FIG. S8. **Reducibility against additional structural parameters for all 60 empirical datasets**. We show the reducibility of each of the 60 empirical datasets against (a) the logarithm of its number of nodes N, (b) the logarithm of its number of hyperedges M, (c) the average hyperedge size (d) the largest order (size - 1) in the hypergraph $d_{\max}$, (e) the average generalized degree, and (f) the degree heterogeneity ratio. Datasets are colored by category. For each metric, we indicate the Pearson correlation coefficient r, its associated p-value, and show the corresponding linear fit (solid line if significant, dashed line otherwise). The reducibility can take very different values even for a fixed value of one of the parameters. For example, sparse hypergraphs (low N/M) take values of χ between 0 and 1. (g) Correlations between all pairs of structural metrics used.

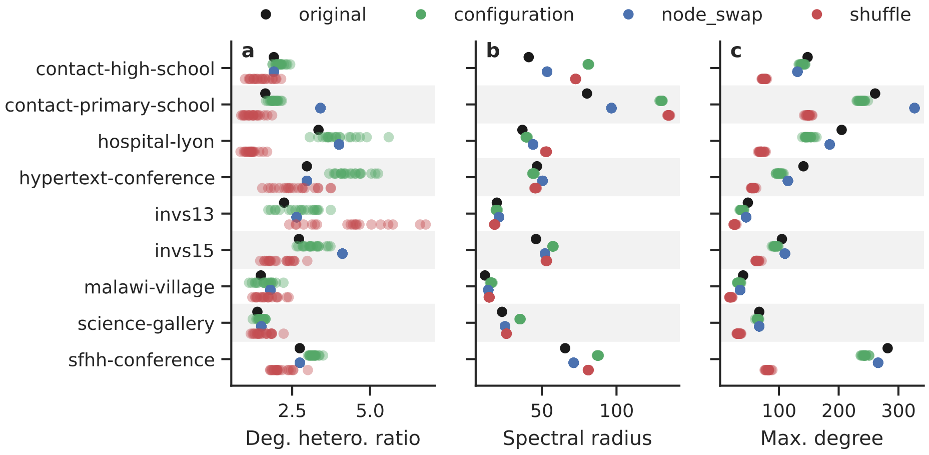

FIG. S9. **Link between reducibility and cross-order degree correlation by randomizing.** We focus on the "contact" datasets because they have high reducibility and nestedness. To each dataset (black), we apply two randomizing strategies: the configuration model (green) and a random shuffling of hyperedges (red). In each case, we show (a) the degree heterogeneity ratio between orders 1 and 2, (b) the spectral radius, and (c) the maximum higher-order degree. Each dot represents one of 20 realizations of each randomizing strategy.

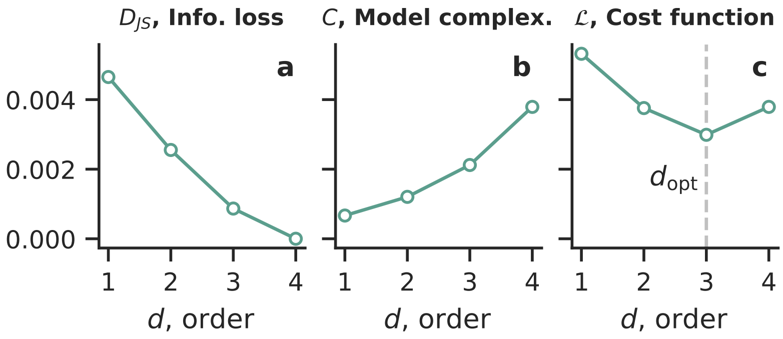

FIG. S10. **The cost function** is the sum of information loss and model complexity based on Jenson-Shannon divergence. We illustrate the terms in the cost function defined in Eq. (4), on an example random simplicial complex: (a) information loss $D_{\mathrm{JS}}(\boldsymbol{\rho}_{\tau}^{[d_{\max}]}|\boldsymbol{\rho}_{\tau'}^{[d]})$, (b) model complexity $\tilde{C}(\boldsymbol{\rho}_{\tau}^{[d]})$, and (c) their sum, the cost function $\mathcal{L}(\boldsymbol{\rho}_{\tau}^{[d_{\max}]}|\boldsymbol{\rho}_{\tau'}^{[d]})$. The minimum of the cost function is indicated by the vertical line. Parameters were set to $N = 100$ nodes and wiring probabilities $p_d = 50/N^d$ at order d with $d_{\max} = 4$. See main text Fig. 2 for results based on Kullback-Leibler divergence.

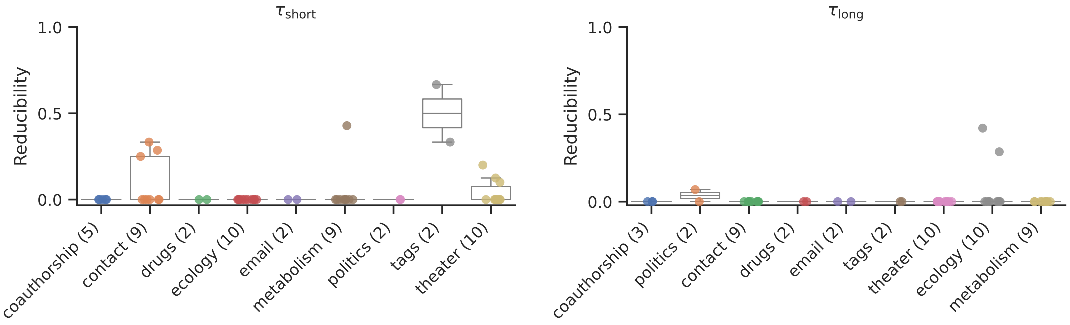

FIG. S11. **Reducibility of empirical datasets using alternative definitions of information loss and model complexity.** We computed it for τ_{short} (left) and τ_{long} (right). Most values are close to zero, at both diffusion times, making this alternate definition less informative in practice.

[1] Y. Zhang, P. S. Skardal, F. Battiston, G. Petri, and M. Lucas, *Deeper but smaller: Higher-order interactions increase linear stability but shrink basins*, Sci. Adv. **10**, eado8049 (2024).
[2] R. M. Gray *et al.*, *Toeplitz and circulant matrices: A review*, Foundations and Trends in Communications and Information Theory **2**, 155–239 (2006).
[3] M. De Domenico and J. Biamonte, *Spectral Entropies as Information-Theoretic Tools for Complex Network Comparison*, Phys. Rev. X **6**, 041062 (2016).
[4] P. D. Grünwald, *The minimum description length principle* (MIT press, 2007).
